# Comparison of radiological and clinical outcomes, complications, and implant removals in anatomically pre-contoured clavicle plates versus reconstruction plates – a propensity score matched retrospective cohort study of 106 patients

**DOI:** 10.1186/s12891-020-03445-5

**Published:** 2020-06-29

**Authors:** Christian X. Fang, Ruiping Liu, Dennis K. H. Yee, Jackie Chau, Tak-Wing Lau, Rebecca Chan, Siu-Bon Woo, Tak-Man Wong, Evan Fang, Frankie Leung

**Affiliations:** 1 Department of Orthopaedics and Traumatology, Queen Mary Hospital, The University of Hong Kong, 102 Pok Fu Lam Road, Hong Kong, China; 2 Department of Orthopaedics, Affiliated Hospital of Nanjing Medical University, Changzhou Second People’s Hospital, Changzhou, 213003 China; 3 Hospital Authority, 147B Argyle Street, Hong Kong, China; 4 David Trench Rehabilitation Center, 1F High Street, Sai Ying Pun, Hong Kong, China; 5 Department of Orthopaedics and Traumatology, Kwong Wah Hospital, 25 Waterloo Road, Mongkok, Hong Kong, China

**Keywords:** Anatomical plate, Locking plate, Clavicle fracture, Plate fixation, Midshaft clavicle

## Abstract

**Background:**

Plate fixation is frequently used to treat displaced midshaft clavicular fractures, however the ideal plate choice remains subject to discussion; reconstruction locking compression plates (RLCPs) are cheaper and can be easily contoured, whereas anatomically pre-contoured locking compression plates (ALCPs) are thought to provide better stability and therefore lower rates of mechanical failure.

To compare the incidence of mechanical failures, functional and radiological outcomes in patients with midshaft clavicular fractures treated with ALCPs versus RLCPs.

**Methods:**

A propensity score matched retrospective cohort study was conducted across two centers. One hundred and six consecutively recruited patients with displaced midshaft clavicular fractures, who were treated with plate fixation and had a minimum follow-up of 6 months, were matched on gender, age, fracture grading, energy of injury, and fracture location. The resulting groups included 53 ALCP-treated fractures and 53 matched controls treated with RLCPs.

**Results:**

During a mean follow-up of 20.5 months, there were no implant deformities in the ALCP group whereas the RLCP group had 6 patients (11.3%, *p* = 0.012) with implant deformities (5 occurrences of plate bending with fracture union, and 1 plate breakage with nonunion). Despite the higher rate of plate deformities in the RLCP group, there were no statistically significant differences in number of patients recovering full shoulder range of motion (ALCP 90.6%, RLCP 88.7%, *p* = 0.751), incidence of rest pain (ALCP 13.2%, RLCP 9.4%, *p* = 0.542), or implant removals (ALCP 49.1%, RLCP 56.6%, *p* = 0.439).

**Conclusion:**

ALCPs may be superior to RLCPs in terms of implant stability but appear to produce similar clinical results.

## Background

Fractures of the clavicle represent 2.6–4% of all fractures in adults, with the majority (69–82%) occurring in the midshaft of the clavicle [1]. Midshaft clavicular fractures are commonly treated nonoperatively with good results [2–6], however plate fixation is indicated when the fracture is severely displaced or causing neurovascular injury. While the precise indications for surgery remain controversial, operative treatment generally results in better early pain control and lower rates of malunion and non-union, albeit at a slightly increased risk of surgical complications [7–10]. Different plate options are available for the fixation of midshaft clavicular fractures, however the ideal plate choice remains subject to discussion.

Traditionally, 3.5 mm reconstruction plates have been used to repair clavicular fractures since they can be easily contoured in two planes to match the S-shaped profile of the clavicle. Satisfactory outcomes have been reported following the use of these implants [11–14]. In addition, angle-stable locking screws have demonstrated improved resistance to pull-out in biomechanical studies [15–19]. Recently, anatomically pre-contoured plates have become more popular and have demonstrated satisfactory outcomes [20–24]. The proposed advantages of pre-contoured plates include improved stiffness, lower profile, and minimal need for additional contouring.

Presently, clinical studies directly comparing anatomical and reconstruction plates are lacking. One recent study of 55 cases reported faster union and better function compared to reconstruction plates, with no mechanical failures in either group [25]. We conducted a retrospective study in a larger cohort of patients and compared patients receiving the two implants using propensity score matching. The objectives of our study were to: (1) compare the radiological outcomes including fracture union and implant stability and (2) compare the incidence of clinically adverse complications and implant removals between anatomically pre-contoured plates and reconstruction plates in midshaft clavicular fractures.

## Methods

**Fig. 1:**
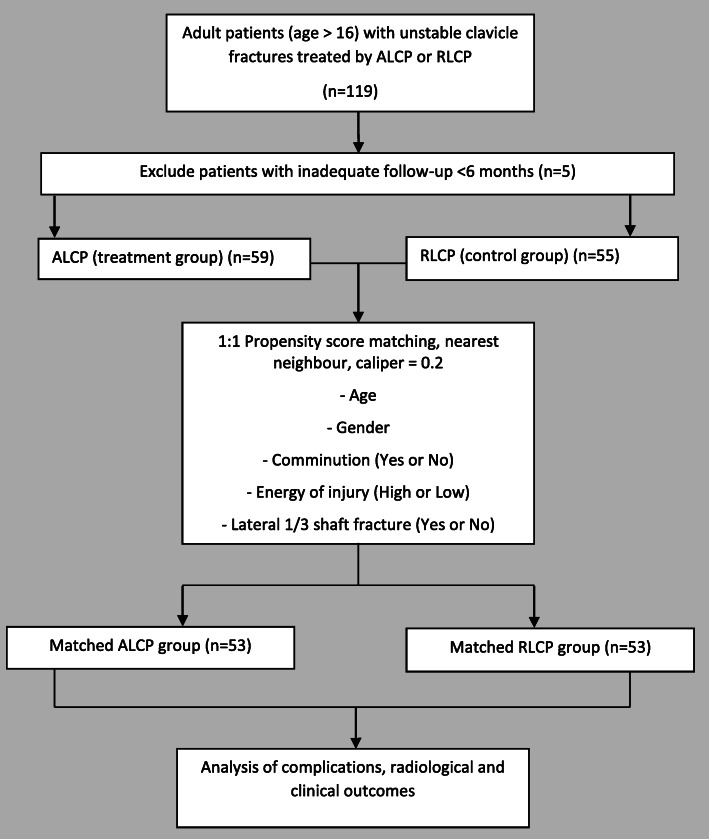
STROBE patient grouping and follow-up flow diagram. Abbreviations: ALCP: anatomic locking compression plate; RLCP: reconstruction locking compression plate

**Fig. 2:**
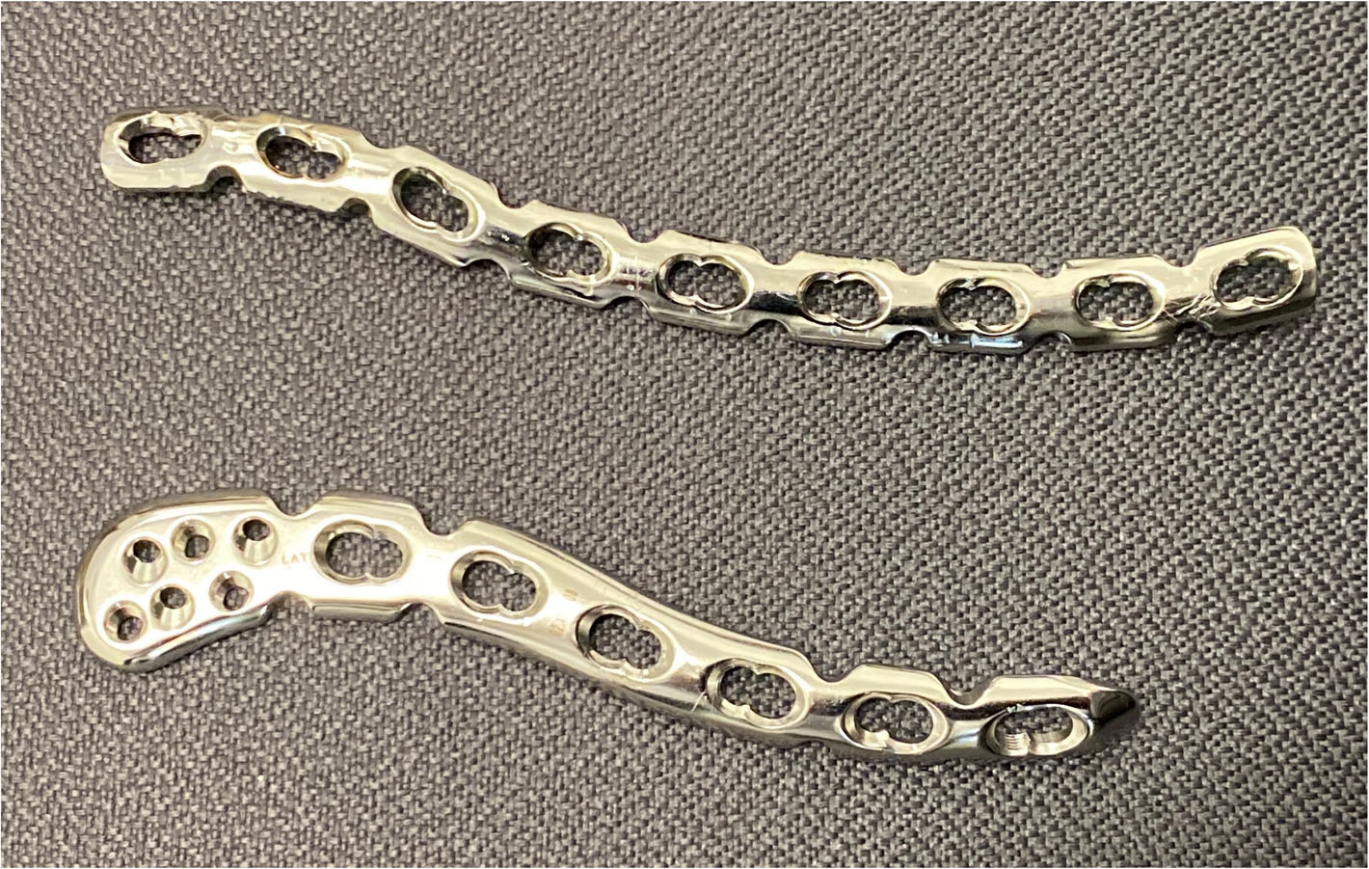
A reconstruction locking plate (above) and anatomic locking compression plate (below) removed from two patients with right-sided clavicle fractures

All consecutive cases coded under the International Classification of Diseases 9th revision (ICD-9-CM) procedure codes 79.19, 79.29, and 79.39 (open or closed reduction of clavicle fractures with internal fixation) across two publicly funded hospitals within a ten-year period were retrospectively identified through a centralized database. This time period represents that in which the ALCP was gradually introduced. Clinical records and radiographs of all patients were evaluated for complications such as implant loosening, implant deformation, defined as more than 5 degree deformity when comparing immediate post-operative and follow-up radiographs, and problems with fracture union. The inclusion criteria were: (1) age greater than 16 years at time of operation, (2) displaced fracture of the clavicle shaft (Robinson [26] type 2B1 or 2B2 and AO/OTA classification [27] type 15.2 A/B/C), (3) fracture operated within 3 months of injury, (4) internal fixation in the superior position with either pre-contoured anatomic locking compression plates (ALCPs) (treatment group) (Stainless Steel 3.5 mm Superior and Superior-Anterior Clavicle Plates, DePuy Synthes, West Chester, PA, USA) or reconstruction locking compression plates (RLCPs) (control group) (Stainless Steel 3.5 mm LCP Reconstruction Plates, DePuy Synthes, West Chester, PA, USA) (Fig. 1). The exclusion criteria were: (1) pathological fractures (excluding osteoporotic) and (2) fracture location lateral to the coracoid. Patients with a follow-up of at least 6 months and those with a known complication prior to 6 months were included for matching and analysis (Fig. 2).

### Operative procedure and rehabilitation

All patients were operated in the supine position under general anaesthesia. A direct approach was used along the clavicle axis or the Langer’s lines. Fixation was performed by anatomical reduction, lag screw and neutralization plate fixation, or by bridging fixation when comminuted. All patients received implants from the same manufacturer. RLCPs were bent in two planes to match the clavicle profile, while ALCPs were only contoured in one plane or by twisting. For both implants, at least two locking or cortical screws were inserted in both ends. Gentle mobilization limited to shoulder level was allowed immediately after operation for the first 6 weeks, followed by full range of motion and strengthening exercises.

### Radiological and clinical outcomes

During follow-up, standard radiographs in AP view were retrieved and examined by two surgeons, who each had at least 10 years of experience, to assess (1) fracture union and (2) complications such as implant loosening, implant deformation and problems with fracture union. Clinically adverse outcomes were recorded and defined as: (1) inability to fully elevate shoulder to 180° or match the contralateral side, (2) presence of any residual pain at rest, (3) reoperations for any reasons including complications and hardware removal, and (4) distal neurological deficits. Unfortunately, functional scores such as the Disabilities of the Arm, Shoulder and Hand (DASH) was not routinely measured in our clinics.

### Propensity score matching

Propensity score matching (PSM) [28] was performed to select cases and match the baseline characteristics of the two groups and minimize confounding from patient selection. Five baseline variables were selected for matching: age, sex, fracture configuration (AO/OTA type A: Simple vs wedge/comminution: types B/C), fracture location (lateral two fifths of the shaft vs middle of shaft or medial shaft) and high-energy injury (such as sports injuries, traffic accidents, and falls from above 2 m). The PSM procedure was carried out using standard nearest neighbour matching, and a caliper value of 0.2 [29] using SPSS software (version 23, IBM, Armonk, USA), R (version 3.10, The R Foundation, Vienna, Austria) and the Thoemmes plugin (version 3.04) [30]. After matching, baseline variables were reported and compared between the groups.

### Statistical analysis

Statistical analysis was performed using SPSS software. Differences between the ALCP and RLCP groups were compared using the Mann-Whitney test. A *p*-value of < 0.05 was considered statistically significant throughout the study. The relative risk, absolute risk reduction and number needed to treat was calculated for outcomes with statistically significant differences.

## Results

Between 2005 and 2015, we obtained 119 patients with unilateral clavicle fractures that fulfilled the inclusion criteria and exclusion criteria. Five patients were lost to follow-up and the remaining 114 had a minimum follow-up of at least 6 months (mean 20.5 months). This included 59 patients with ALCPs and 55 patients with RLCPs. After propensity score matching, there were 53 patients in each group. No baseline factors had statistically significant differences between groups before (Mann-Whitney test, *p* ≥ 0.300) or after matching (Mann-Whitney test, *p* ≥ 0.317). The maximum standardized mean difference was reduced from 0.162 before matching to 0.119 after matching. Table 1 shows the baseline patient characteristics for both groups before and after matching.

**Table 1:** Baseline variables of patients before and after matching

	Before Matching						After Matching					
	ALCP (*n* = 59)		RLCP (*n* = 55)		Std Mean Diff^a^	*p* value*	ALCP (*n* = 53)		RLCP (*n* = 53)		Std Mean Diff^a^	*p* value*
Mean Age (Mean, (range))	41.4	(16–71)	42.5	(18–84)	0.068	0.779	40.7	(16–71)	42.1	(18–77)	0.091	0.620
Males	80%	(47)	78%	43	−0.036	0.847	79%	(42)	77%	(41)	−0.045	0.815
Lateral 2/5 shaft fractures	19%	(11)	13%	(7)	−0.162	0.389	13%	(7)	13%	(7)	0.000	1.000
AO Type 15.2 B/C: Wedge or comminuted fractures	64%	(38)	71%	(39)	0.138	0.461	64%	(34)	70%	(37)	0.119	0.537
High energy injury	58%	(34)	55%	(30)	−0.062	0.741	53%	(28)	55%	(29)	0.037	0.846
Operated by specialist with experience above 6 years^b^	92%	(54)	93%	(51)	0.044	0.813	91%	(48)	92%	(49)	0.067	0.729
Concomitant fractures^b^	12%	(7)	18%	(10)	0.177	0.346	11%	(6)	17%	(9)	0.162	0.405
Open fracture^b^	0%	(0)	1.8%	(1)	0.270	0.300	0%	(0)	1.8%	(1)	0.275	0.317
ASA 1/2/3^b^	(36/19/4)		(30/21/4)			0.512	(33/17/3)		(29/21/3)			0.467

^a^Std. Mean difference = Mean difference / Mean standard deviation

^b^Factors not included in PSM

*Mann-Whitney test

Out of the 53 matched patient pairs in the ALCP and RLCP groups, there were no significant differences in clinical outcomes. Forty-eight (90.6%) ALCP and forty-seven (88.7%) RLCP patients achieved full range of motion of relative to the contralateral shoulder (Mann-Whitney test *p* = 0.751). Seven (13.2%) ALCP and five (9.4%) RLCP patients experienced some degree of chronic pain at rest (Mann-Whitney test *p* = 0.542). Twenty-six (49.1%) ALCP and thirty (56.6%) RLCP patients had implant removals (Mann-Whitney test, *p* = 0.439).

**Fig. 3:**
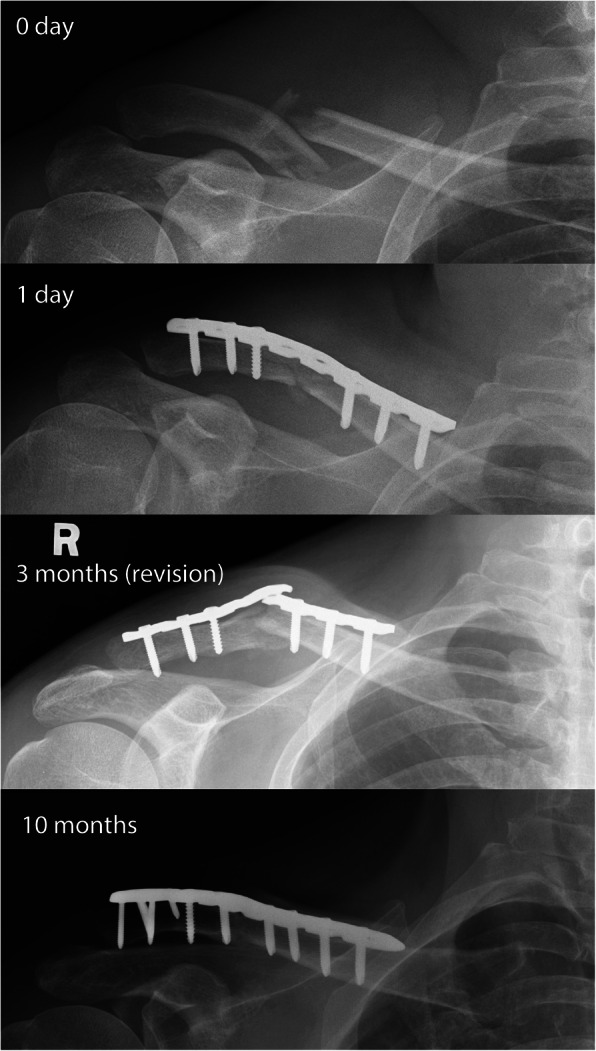
A patient with a comminuted shaft fracture treated with an RLCP. At three months, the implant had broken and the fracture displayed non-union. The patient continued to have mechanical pain and crepitation upon movement. The fracture healed after revision with an ALCP and bone grafting

Six (11.3%) patients in the RLCP group were observed to have post-operative implant deformation during the 0–3 months follow-up, versus none (0%) in the ALCP group (Mann-Whitney test, *p* = 0.012). Out of these six patients, five had fracture union despite progressive angulation of the reconstruction plate. One patient had implant breakage and non-union after RLCP and was re-operated after 3 months with bone grafting and revision plating using an ALCP (Fig. 3).

**Fig. 4:**
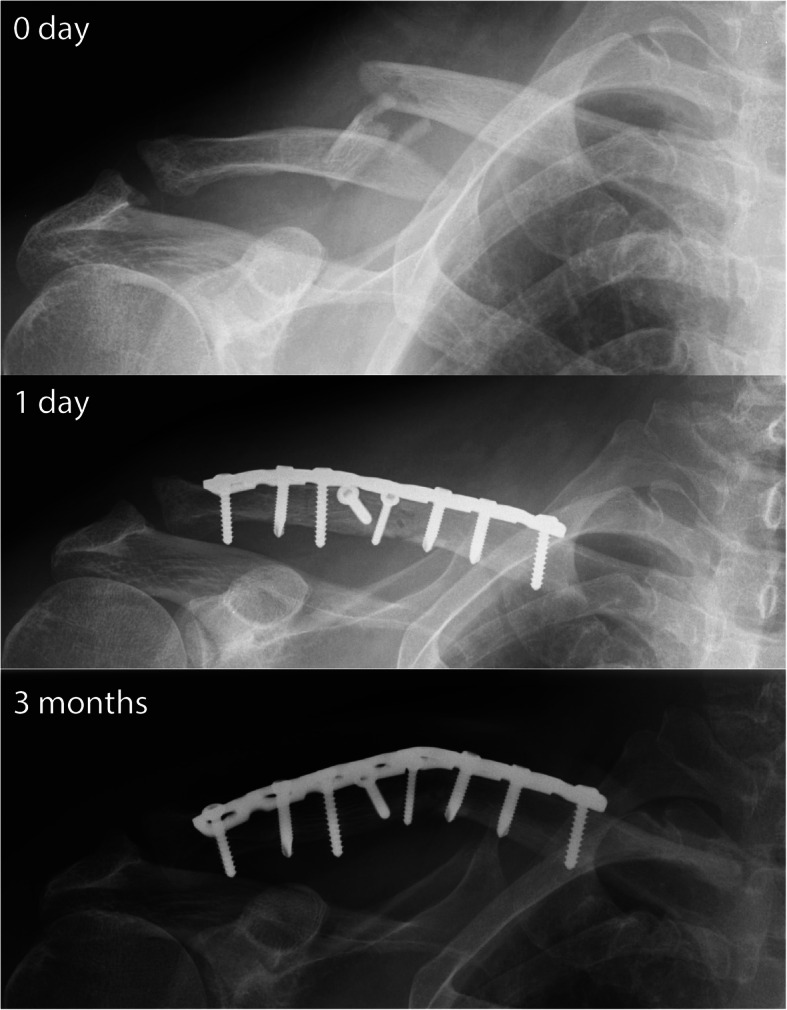
A patient with a comminuted midshaft fracture, treated by lag screws and an RLCP. At three months, the fracture united with an angulated implant and a visible bump. Despite this, the patient was pain-free with full functional recovery and declined implant removal

Out of the six patients with plate deformations, three had comminuted fractures (Fig. 4), one fracture was located at the lateral third, and two suffered from a high-energy injury mechanism. These numbers were not sufficient for a statistically meaningful subgroup analysis of predisposing risk factors other than implant type. Screw pull-out was not observed as a mechanism of failure in any patients.

One patient treated with ALCP had iatrogenic injury to the brachial plexus with median nerve paraesthesia that persisted beyond 2 years. There were no patients with re-fractures or wound infections. Up to the latest follow-up, all patients had their clavicle fracture completely united, including the patient with non-union who received reoperation.

With RLCP being the risk-exposed group, the relative risk (RR) for implant deformation was 7.69%. The absolute risk reduction (ARR) was 11.3% and the number needed to treat (NNT) was 9.0. The combined incidence for any suboptimal clinical events including re-operations for removals was 54.7% (29 events) for the ALCP group and 69.8% (37 events) for the RLCP group (Mann-Whitney test *p* = 0.111). Table 2 displays a list of all outcomes before and after PSM.

**Table 2:** Outcome variables of patients before and after matching

	Before Matching					After Matching				
	ALCP (*n* = 59)		RLCP (*n* = 55)		*p* value*	ALCP (*n* = 53)	RLCP (*n* = 53)			*p* value*
Unable to regain full range of motion	12%	(7)	12.7	(7)	0.889	9.4%	(5)	11%	(6)	0.751
Persistent pain at rest	14%	(8)	11%	(6)	0.668	13%	(7)	9.4%	(5)	0.542
Reoperation for hardware removal	48%	(28)	58%	(32)	0.254	49%	(26)	57%	(30)	0.439
Reoperation for complications	0%	(0)	3.6%	(2)	0.141	0%	(0)	1.8%	(1)	0.317
Implant failures	0%	(0)	11%	(6)	0.009	0%	(0)	11%	(6)	0.012
Infection	0%	(0)	1.8%	(1)	0.300	0%	(0)	0%	(0)	1.000
Brachial plexus injury	1.8%	(1)	0	(0)	0.334	1.8%	(1)	0%	(0)	1.000
Adverse outcomes of any kind above	54%	(32)	71%	(39)	0.068	55%	(29)	70%	(37)	0.111

*Mann-Whitney test

## Discussion

Clavicle fractures are one of the most common orthopedic injuries, with a bimodal peak incidence occurring in youth and the elderly [31]. Although most patients can expect good outcomes with conservative treatment [2–6], recent studies suggest that internal fixation results in earlier rehabilitation with better rates of successful bone union [7–10]. However, it does bear the increased risk of surgery-related complications [14]. Surgical treatment is generally indicated in cases with shortening > 2 cm, skin impingement, or painful non-union, however the precise set of indications remains disputed [8], as does the ideal plate choice for surgery. Our study sought to compare the incidence of mechanical failures, removals, functional and radiological outcomes in patients with midshaft clavicular fractures treated with ALCPs versus RLCPs.

In terms of structural features, the two plate types used in our study can be compared as follows: the RLCP is a straight plate made of low-stiffness, annealed metal which facilitates manual contouring (reconstruction), whereas the ALCP is made of high-stiffness, cold-worked metal which is pre-shaped to match the S-shaped profile of the clavicle. Both use angle-stable locking screws for better pull-out resistance [15–18], with the ALCP also accommodating optional smaller screws for fixation at the distal end.

Biomechanical studies have demonstrated a considerable advantage in plate stiffness for ALCPs compared to RLCPs. The average cantilever failure load observed (40-42 N) for reconstruction plates in one study [16] was only a quarter of that observed (170-184 N) for cold-worked plates in a separate study by the same authors [15]. When tested in the tension band mode under optimal positioning, the RLCP constructs started to fail at significantly lower forces than the conventional plates (251 N–355 N vs 300 N–345 N) [15, 16]. ALCPs have also been shown to withstand over three times the force in axial loading (1790 N/mm vs 5740 N/mm) and over twice as much torsion (130 Nm/mm vs 300 Nm/mm) [32]. Failure by screw hole fracture typically occurs at a similar load (about 450 N) for both implants [33]..

RLCPs may be mechanically inferior to ALCPs when used to treat comminuted clavicle fractures. Taylor used a 3D mathematical model to demonstrate that the clavicle can withstand a combination of bending and torsional forces in the X, Y, and Z axes [34]. This supports the disputed claim that the clavicle does not have a true “tension-side”, and therefore the tension band effect may not work for simple fractures, requiring the use of a sturdier plate. Finite element analysis has shown that anatomical plates may significantly reduce local stress under complex loads when compared to reconstruction plates [35]. These results suggest that RLCPs may not be able to withstand physiological stresses as well as ALCPs when used as a bridge in comminuted fractures.

To our knowledge, ours is one of the largest comparative clinical studies between ALCPs and RLCPs for the treatment of unstable clavicle fractures. While our study was not a randomized control trial (RCT), we obtained two reasonably balanced treatment groups using PSM [29]. This technique attempts to approximate an RCT by matching multiple confounding variables between groups, thereby minimizing bias and increasing the validity of the results. The drawbacks of PSM include the need for larger samples, a reduction in statistical power, and the risk of overlooking important confounders at the planning stage.

The deformation rate of reconstruction plates in our study (11.3%) was similar to those reported by other clinical studies: Liu (*n* = 59, 8.5%) [36], Shin (*n* = 125, 8%) [12], Woltz (*n* = 112, 12.6%) [13], Shen (*n* = 232, 14%) [14] and Virtanen (*n* = 28, 3.4%) [10]. Additionally, our observed incidence of mechanical failures (0%) among the anatomically pre-contoured plates is also consistent with studies by Campochiaro (*n* = 89, 2%) [20], Fridberg (*n* = 105, 5%) [21], Hundekar (*n* = 20, 0%) [22], Ranalletta (*n* = 72, 3%) [23] and Robinson (*n* = 95, 1%) [7].

Our observed implant removal rates (49% of ALCPs and 57% of RLCPs) are comparable to the results of studies by Schemitsch (*n* = 153, 38% removed of mixed implant types) [37] and VanBeek (*n* = 42, 64% non-anatomical and 11% anatomical plates removed, but with shorter mean follow-up in the anatomical plate group) [24]. It is worth noting that Schemitsch also found shorter body height (< 175 cm) to be a risk factor for implant removal. The most common indication for implant removal in our clinics is discomfort from implant impingement. This may be subject to regional and cultural beliefs and the fact that public healthcare coverage in our region minimizes the cost of implant removal. Anterior-inferior positioning and the use of lower-profile 2.7 mm plates may result in lower rates of removal [38].

RCLPs are designed with indented edges which reduce the cross-sectional moment for sideways contouring. In contrast, ACLPs have a smoother edge and may produce less soft tissue irritation. Unfortunately, cadaver studies have demonstrated that “anatomically fitting” plates do not actually fit the bone in 5–32% of the population [39, 40]. This is especially true in women, whose clavicles are shorter and display more exaggerated curvature [41]. This is consistent with our experience that ALCPs nearly always require some degree of additional contouring.

The limitations of our study include the retrospective design, possibility of selection bias despite matching, lack of functional outcome scores, non-standardized and lack of documenting of removal indications, not comparing the speed-of-union as some studies did using CT scans, and having only a moderate sample size of 106 cases.

## Conclusions

1. We recommend avoiding use of RLCPs for less stable comminuted fractures as ALCPs provide better mechanical stability. Patients treated with reconstruction plates should therefore adopt a less aggressive rehabilitation programme.
2. The higher observed incidence of implant plastic deformation for reconstruction plates did not translate to inferior clinical results in terms of union, pain and range of motion.
3. The two treatment groups had similar incidences of implant removal. Patients receiving ALCPs should expect similar risk of soft tissue discomfort leading to the need for later removal.

## Data Availability

The data that support this study are available from the Hong Kong Hospital Authority Clinical Data and Reporting System (CDARS), but restrictions apply to these data, which were used under license for the current study, and so are not publicly available. Data however are available from authors upon reasonable request and with permission of Dr. Christian Fang.

## Abbreviations

RLCP: Reconstruction locking compression plate
ALCP: Anatomically pre-contoured locking compression plate
ICD-9-CM: International Classification of Diseases, 9th Revision, Clinical Modification
AO/OTA: AO-Müller/Orthopaedic Trauma Association
DASH: Disabilities of the Arm, Shoulder and Hand
PSM: Propensity score matching
RR: Relative risk
ARR: Absolute risk reduction
NNT: Number needed to treat
RCT: Randomized controlled trial
CT: Computed tomography
